# Supplementary material for: Patterns of Intron Gain and Loss in Fungi
Source: PLoS Biol. 2004 Nov 30;2(12):e422. doi: 10.1371/journal.pbio.0020422 (PMC532390; doi:10.1371/journal.pbio.0020422)
Supplement: Table S1 — Also available at http://genes.mit.edu/NielsenEtAl/. (4.3 MB ZIP). [file pbio.0020422.st001.zip › NielsenEtAl/html/660.html]

AN3221.1.NCU05519.1.MG07843.1.FG11084.1


```
 CLUSTAL W (1.82) Multiple Sequence Alignments - Introns Inserted


Sequence 1: AN3221.1	510 aa
Sequence 2: MG07843.1	521 aa
Sequence 3: FG11084.1	499 aa
Sequence 4: NCU05519.1	519 aa
Alignment Length: 521 aa
Number Identitical Residues: 234 aa
Alignment Score (without introns) 12500


MG07843.1 	MASNMDEKVSKDVGVEHGSSSEDRIQTAAIMNDDTLRQAAARGQLATDA~YGNPVLQFDP
NCU05519.1	MAAGVSETMTSEKRRQSLSESD--TKEGYFENAPGAHYRAERGQAATDI2HGNPLVELDP
FG11084.1 	MES----------------SN----HNEKFQNDTASNSDAAGG--AVET2VENAQFYIDP
AN3221.1  	MAT-------TSVADEKAMSG----HQENLKPVDDPTQFAHRGQAATDQ~YGHALFEFDK
          	* :       ..   .   ..    :   :         *  *. *.:    :. . :* 

MG07843.1 	KAVRKLRMKMDLYLVPTVALLYLFCFIDRANIG1NARIAGLEKDLGLTGNDYNIILSVFY
NCU05519.1	VAETKLRRKIDLYVVPTVAILYLFCFIDRANIG1NARLDKLEKDLDLHGYDYNALLSVFY
FG11084.1 	KQEAKLRRKIDFMIVPTVCLLYLFCFIDRANIG1NARLAGLEEDLGMKGSDYNLVLSTFY
AN3221.1  	KAESRLRWKMDFYLLPTVALLYLFCFIDRANIG1NARLAGFDTDLGLKGNDYNKVLSIFY
          	    :** *:*: ::***.:************* ***:  :: **.: * *** :** **

MG07843.1 	ISYIIFEIPATVLCKWMGPGWFLPATSLLFGIVSIATA~FVHDRAAICGVRFLLGIFEAG
NCU05519.1	VGYIVFEIPANIMCKWMGPGWFLPLTSLGFGIMSVCMA~FVNNFSQACGVRFLLGVFEAG
FG11084.1 	ISYIIFEIPANLLCKYMGPGWFIPLTSLGFGLVSIFTA~FVHNVPQICGVRFVLGIFEAG
AN3221.1  	VSYILFEIPCNLCCKWMGPGYFLPLSTLCFGAASVATA2FVDNIQEASGVRFVLGIFEAG
          	:.**:****..: **:****:*:* ::* **  *:  * **.:    .****:**:****

MG07843.1 	MLPGIAYYLSRWYQRAELAFRLSLYMVMAPLAGAFGGLLASAILTLDHFGSIHSWRMIFA
NCU05519.1	MLPGIAYYLSRWYRRSELTLRLSLYIVMSPLAGAFGGLLASGILKLDHFGSLHGWRMIFG
FG11084.1 	MLPGIAYYMSRWYRRSELTFRLSLYIVMAPLAGAFGGLLASAILKLPNFGRFKSWEMIFA
AN3221.1  	MLPGIAYYLSRWYRRSELAFRLALYIVMAPLAGAFGGLLASAILTLDHFGSLHTWRMIFA
          	********:****:*:**::**:**:**:************.**.* :** :: *.***.

MG07843.1 	~IEGIVTIIISIIAFF~TLTDRPETARWMTEEEKILCEARVKSERVGQTE~VLDKMDMKK
NCU05519.1	~IEGIITVGLSLIGFL~TLTDHPATAKWLSQEEKDLAIARVKSERIGQTE~IIDKMDAKK
FG11084.1 	1IEGIVTVVLSLIAFA1TLTDRPDTARWLTEEERALAINRIKAERLGTTE~VLDKMDKAK
AN3221.1  	~IEGIVTCALGLIAFL~TLTDRPETARWLSQEEKDLAIARLKSERVATTE0VLDKIDTKK
          	 ****:*  :.:*.*  ****:* **:*:::**: *.  *:*:**:. ** ::**:*  *

MG07843.1 	LKRGMTNPVTLQIAFVFLCNNVTVQGLAFFLPTIVSTIYPTFSTVQKQLYSVPPYVVGAF
NCU05519.1	LKRGIFNPVVFFTGFAFLLNNITVQGLAFFAPTVVATIYPTKNIIQKQLLTVPPYIVGGF
FG11084.1 	IFAGIRSPLTLSTSIIFLLDNITVQGLAFFLPTIVRTIYPDRSTISQQLFTVPPYVVGAF
AN3221.1  	LLRGIFSPVTLSTSFIFLLNNITVQGLAFFAPTIVRTIYPDASVVSQQLHTVPPYVVGAF
          	:  *: .*:.:  .: ** :*:******** **:* ****  . :.:** :****:**.*

MG07843.1 	FTVAFPSLSWYLDRRQFLIALSAPMVMTGYIMFLASKVATVRYAATFLIASSAMVLGPMS
NCU05519.1	FTVLMPLISRWLDRRQIIIIACCPLVMVGYIMFLATENAHVRYGAAFLVSTSVFAAGPLT
FG11084.1 	FTVLIPALSYKMDRRQIFIILCAPLVMVGYAIFLGTNNGQARYGATFLVASSAFALAPLT
AN3221.1  	FTLLFPFLSWRFDKRMPFFIAGPPLMMIGYIMFLASSDAMTRYGATFLIASGAFAFGALC
          	**: :* :*  :*:*  ::    *::* ** :**.:. . .**.*:**:::..:. ..: 

MG07843.1 	NAHISANVVSDTARSSAIGLN0V~MFGNVGGLISTWSFLPWDGPDYKIGNGLNLATSSMI
NCU05519.1	NSQVSANVVSDTARSSAIAYN~V1MLGNVGGLVATWSFLPFDAPKYHIGNGINLAASGGT
FG11084.1 	NSHISANVLSDTARSAAIGTN~V1MFGNIGGLIATWSFVKSDGPDYPIGNGLNLATSSTI
AN3221.1  	NAHVSNNVISDTARSAAIGTN~V~MFGNIGGLVSTWSFLQFDGPDYHIGNGLNLATSSMC
          	*:::* **:******:**. * * *:**:***::****:  *.*.* ****:***:*.  

MG07843.1 	LIVSTGALFWMNWDNKRREKLDVDKELSGLSQQEIEDLDWKHPSFRWRP
NCU05519.1	LIIALCLLIWMKRDNNKRELRNAEEELTGMSQQEIQDLDYKHPGFRWKP
FG11084.1 	LVLGTLTLLWIKRDNQKRANRSAEEELAGMSQQQIQDLDWKNPNFRWIA
AN3221.1  	LILSALLWMWMKWDNKKRDDTDVDAALDGLTQKQVQDLDWRNPAFKWRP
          	*::.    :*:: **::*   ..:  * *::*::::***:::* *:* .
```
